# Hospitalization Risk Due to Respiratory Illness Associated with Genetic Variation at *IFITM3* in Patients with Influenza A(H1N1)pdm09 Infection: A Case-Control Study

**DOI:** 10.1371/journal.pone.0158181

**Published:** 2016-06-28

**Authors:** Vânia Gaio, Baltazar Nunes, Pedro Pechirra, Patrícia Conde, Raquel Guiomar, Carlos Matias Dias, Marta Barreto

**Affiliations:** 1 Departamento de Epidemiologia, Instituto Nacional de Saúde Doutor Ricardo Jorge, 1649–016 Lisboa, Portugal; 2 Departamento de Doenças Infeciosas, Instituto Nacional de Saúde Doutor Ricardo Jorge, 1649–016 Lisboa, Portugal; University of Pittsburgh, UNITED STATES

## Abstract

**Background:**

Recent studies suggest an association between the Interferon Inducible Transmembrane 3 (*IFITM3*) rs12252 variant and the course of influenza infection. However, it is not clear whether the reported association relates to influenza infection severity. The aim of this study was to estimate the hospitalization risk associated with this variant in Influenza Like Illness (ILI) patients during the H1N1 pandemic influenza.

**Methods:**

A case-control genetic association study was performed, using nasopharyngeal/oropharyngeal swabs collected during the H1N1 pandemic influenza. Laboratory diagnosis of influenza infection was performed by RT-PCR, the *IFITM3* rs12252 was genotyped by RFLP and tested for association with hospitalization. Conditional logistic regression was performed to calculate the confounder-adjusted odds ratio of hospitalization associated with *IFITM3* rs12252.

**Results:**

We selected 312 ILI cases and 624 matched non-hospitalized controls. Within ILI Influenza A(H1N1)pdm09 positive patients, no statistical significant association was found between the variant and the hospitalization risk (Adjusted OR: 0.73 (95%CI: 0.33–1.50)). Regarding ILI Influenza A(H1N1)pdm09 negative patients, CT/CC genotype carriers had a higher risk of being hospitalized than patients with TT genotype (Adjusted OR: 2.54 (95%CI: 1.54–4.19)).

**Conclusions:**

The *IFITM3* rs12252 variant was associated with respiratory infection hospitalization but not specifically in patients infected with Influenza A(H1N1)pdm09.

## Introduction

Influenza is a contagious and debilitating disease of the respiratory tract that can lead to hospitalization and even death, remaining a public health threat. In April 2009, an outbreak of influenza A(H1N1)pdm09 infection was detected in Mexico, with subsequent cases observed in many other countries, including Portugal. It has spread quickly throughout the world and caused at least 15000 deaths in less than a year [1]. Unlike the disease pattern observed during epidemics of seasonal influenza, many of the severe cases of influenza A(H1N1)pdm09 virus infection were observed in healthy young people, with approximately 90% of all deaths due to influenza occurring in those younger than 65 years [2]. Most people infected with influenza A(H1N1)pdm09 virus experienced mild disease, with upper respiratory illness similar to seasonal influenza virus infection. In contrast, gastrointestinal symptoms occurred more frequently in patients infected with pandemic influenza virus than in those with seasonal influenza [3]. According to the Portuguese Influenza Surveillance Program (NISP), 64% of the laboratory-confirmed cases for the influenza A(H1N1)pdm09 virus were detected in the age group of 5–14 years old. A total of 1436 hospitalizations were reported, and the estimated mortality rate was 1.17 per 100000 inhabitants, corresponding to 124 laboratory confirmed influenza deaths [4].

It is commonly accepted that the severity of influenza A(H1N1)pdm09 infection results from a complex interplay between host and pathogen factors but there are many gaps in the basic understanding of what influences illness severity mainly among people without known comorbidities. While some individuals resist infection or recover quickly, others experience severe disease associated with the infection. Intensive research has been performed on the virulence of the virus including its genomic variability but very little is known about the host genetic background influence in the influenza outcome, despite its crucial impact on the immune response and the course of infection [5]. Consequently, in 2009, the World Health Organization identified studies of the host genetic factors’ role on susceptibility to severe influenza as a priority [6].

Although characteristics such as age, comorbidities, degree of pre-existing immunity, immunosupression, pregnancy and smoking influence the acquisition, progression and resolution of influenza infections, genetic variants found in human populations affect the specificity of virus binding and subsequent effectiveness of the immune response [7]. Their importance in humans has already been shown for several bacterial and viral pathogens [8] and it is currently known that infectious disease lethality has a heritable component [9]. In addition, animal models seem to support the biologic plausibility of a genetic susceptibility to influenza A virus, given that some mouse strains are more susceptible to influenza virus infection and severity, independently of virus subtype, suggesting common infection pathways [10].

It has been shown that an interferon inducible transmembrane 3 protein (IFITM3), which acts as a viral restriction factor that mediates cellular resistance to several viruses by blocking early stages of viral replication, profoundly alters the course of influenza virus infection in a knockout mouse model. Mice lacking a functional *Ifitm3* gene developed severe viral pneumonia when challenged with normally low pathogenic viruses, and protection was re-established with reintroduction of *Ifitm3*. In humans, an overrepresentation of individuals with the *IFITM3* rs12252 variant (C allele) that alters a splice acceptor site was found in European patients who required hospitalization as a result of influenza infection [11].

Recent studies suggest a close association between the *IFITM3* rs12252 variant (C allele) and influenza severity infection. [12,13]. More specifically a meta-analysis which included the previously referred studies suggests a significant association between the *IFITM3* rs12252 variant (C allele) and severe influenza susceptibility, but not in mild influenza subjects, both in UK Caucasians and Han Chinese populations, concluding that the C allele constitutes a risk factor for more severe influenza infection cases [14].

Considering the wider confidence intervals, the small samples sizes and the heterogeneity in the inclusion criteria of the previous studies, the main objective of the present study was to estimate the association between the *IFITM3* rs12252 variant (C allele) and the risk of hospitalization due to respiratory illness in Portuguese patients with influenza A(H1N1)pdm09 infection using an appropriately designed epidemiological study.

## Methods

### Study design and participants

A case-control genetic association study, comparing the frequency of *IFITM3* rs12252 variant between hospitalized influenza-like illness (ILI) patients (cases) and non hospitalized ILI patients (controls) was performed. Biological samples from cases and controls consisted on the nasopharyngeal/oropharyngeal swabs received by the Portuguese National Influenza Reference Laboratory, at the National Health Institute Doctor Ricardo Jorge (INSA) for diagnostic purposes during the H1N1 pandemic, from the Portuguese Laboratory Network for the Diagnosis of Influenza Infection between September 2009 and February 2010 [15].

INSA coordinated this network that was responsible for carrying out the laboratory diagnosis of influenza A(H1N1)pdm09 infection requested by the National Health Service during the pandemic (H1N1)2009. This network received swabs from ILI patients identified by primary healthcare units and hospitals. All information collected regarding clinical and epidemiological information, such as patients’ demographic data, signs and symptoms and underlying conditions and laboratory results were stored in a national common database. A total of 62089 ILI cases were reported to the laboratory network during the H1N1 pandemic. From these 25985 were laboratory-confirmed influenza cases, over 99% of which were associated with the new pandemic strain A(H1N1)[15]. At INSA 16376 samples for Influenza diagnosis were received. Of those, 7780 influenza A(H1N1)pdm09 positive cases were identified.

An ILI positive patient was defined as a patient presenting signs and symptoms complying to the European ILI case definition [16], who was swabbed and tested positive for influenza A(H1N1)pdm09 using real-time polymerase chain reaction (RT-PCR) [17]. ILI negative patients were ILI patients who were swabbed and tested negative for the Influenza A(H1N1)pdm09 virus. ILI patients tested negative for Influenza A(H1N1)pdm09 virus also tested negative for the Influenza A and B virus subtypes.

All included patients were selected from the INSA cases, whose tests for Influenza diagnosis were performed at National Influenza Reference Laboratory (INSA). The following exclusion criteria were used to perform the selection: (1) patients over 65 years old; (2) immunodepressed or transplanted patients (3) patients with chronic diseases (diabetes, lung, kidney, cardiovascular, liver, neurological, immunologic and oncologic diseases); (4) pregnant women; (5) notification data before the mitigation phase (01-09-2009); (6) patients for whom the time lapse between the symptoms onset and the sample collection was over 7 days, to avoid misclassification due to low viral load of the sample collected; (7) samples with unavailable information about hospitalization; (8) samples with unavailable laboratorial result for A(H1N1)pdm09 virus; (9) samples stored outside of the National Health Institute Doutor Ricardo Jorge.

**Fig 1:**
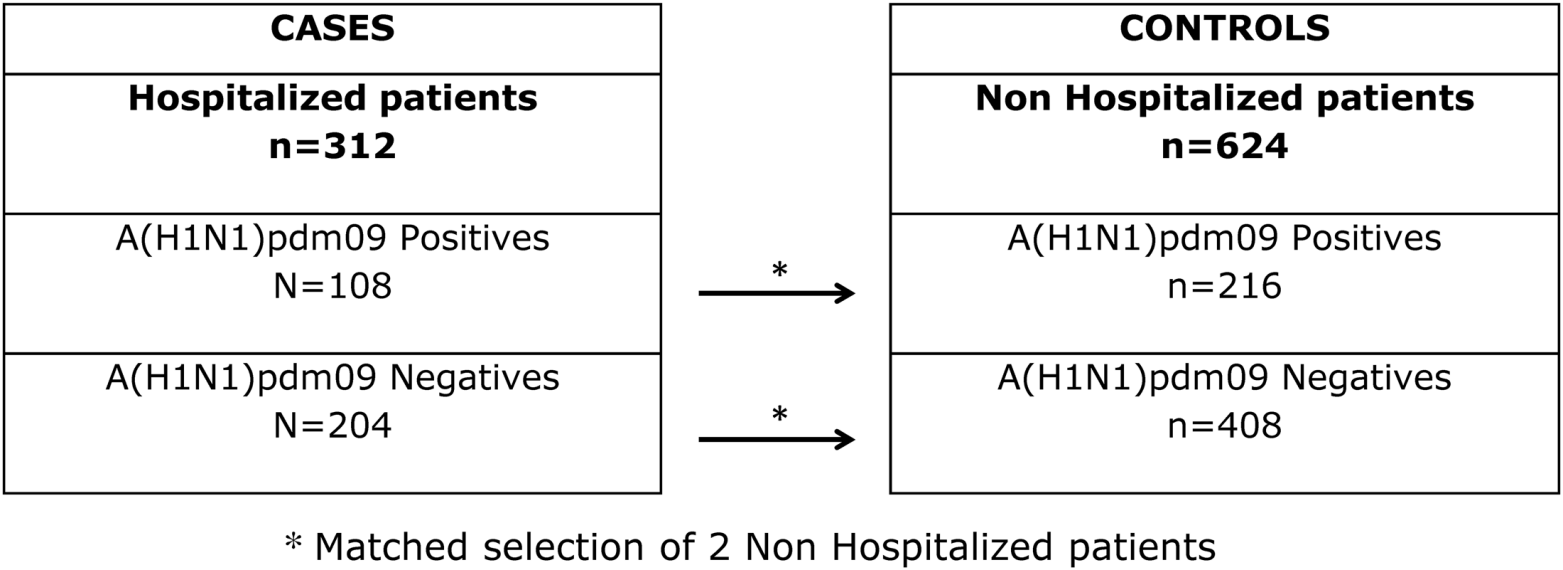
Schematic representation of the sampling process.

After the exclusion criteria application, 312 hospitalized patients were identified. These cases were stratified in two groups: hospitalized Influenza A(H1N1)pdm09 positive cases (n = 108) and hospitalized Influenza A(H1N1)pdm09 negative cases (n = 204). Since the risk of developing Influenza varies with time and to assure that the selected control group was at similar risk of developing the health outcome of interest, for each case in the group of hospitalized cases, 2 non-hospitalized controls matched for the week of onset of symptoms were selected from the Portuguese Laboratory Network database (Fig 1). Hospitalization was used as a measure of disease severity. In this context, it was defined as a hospital admission due to ILI complications with a concomitant hospital stay for more than twenty-four hours. The study protocol was approved by the Ethics Committee of the National Health Institute Doutor Ricardo Jorge. Since this study used irreversibly anonymized samples from the NPIS, obtaining informed consent from the participants was impossible and therefore the study was authorized by the Ethics Committee of the National Health Institute Doutor Ricardo Jorge without informed consent by the participants.

### Laboratory diagnosis

For laboratory diagnosis and further virological characterization, the nasopharyngeal/oropharyngeal swabs were collected into a suitable transport medium for preservation of virus, molecular diagnostic and virus culture. Real-time reverse transcriptase PCR methodologies and platforms were used for laboratory diagnosis of influenza infection [17]. Additional diagnoses, viral isolation, antigenic and genetic characterization of viral isolates were performed according to the procedures previously described [15]. During the 2009/10 influenza season, the laboratory network just carry out the laboratory diagnosis of influenza A(H1N1)pdm09 infection requested by the National Health Service and the samples were only tested for the influenza A(H1N1)pdm09, influenza A(H1), influenza A(H3) and influenza B virus.

### Genotyping

Genomic DNA was extracted from the nasopharyngeal/oropharyngeal swabs using an automatic system (MagNA Pure LC—Roche) with the MagNA Pure LC Total Nucleic Acid Isolation Kit. The rs12252 SNP in the *IFITM3* gene was genotyped by restriction fragment length polymorphism (RFLP) using the following forward and reverse primers: TGAGGGTTATGGGAGATGGGGT (F) and GGAGAGGAGATGGTGAGGGGA (R) in standard PCR conditions. The restriction enzyme *MscI* was used to cut the PCR product in the presence of the wild type T allele and the originated fragments were visualized on a 2% agarose gel.

### Statistical analysis

Statistical analysis was performed using the R program [18]. *P-values*<0.05 were considered to be statistically significant. The T-test and the Wilcoxon test were used to access differences of quantitative variables according to their adherence to the normal distribution. Proportions were compared using the Pearson's chi-squared test.

Regarding age and sex, the National Hospitalization Discharge (NHD) database was used to assess if the sample of influenza positive hospitalized cases analyzed in the present study was a representative of the total influenza (International Codifications of Diseases code 487.0) hospitalized patients in the NHS, during the same period of time and excluding patients over 65 years, with chronic diseases and pregnant women. Allelic and genotypes frequencies were obtained by direct count and were tested for the Hardy-Weinberg Equilibrium, using the Hardy Weinberg R package [19]. To perform the case-control association analysis, hospitalized patients were considered the cases and non-hospitalized patients were considered the controls. The analysis was stratified by ILI A(H1N1)pdm09 positive and negative patients. To estimate the Adjusted *Odds-Ratio*, we used Conditional Logistic Regression for matched Pairs Data including the potential confounding effect of age and gender variables.

## Results

From the 936 patients selected, 909 had sufficient available frozen swabs for DNA extraction. We successfully genotyped 792 samples: 268 from ILI A(H1N1)pdm09 positives patients (84 hospitalized cases and 184 non-hospitalized controls) and 524 from ILI A(H1N1)pdm09 negatives patients (173 hospitalized cases and 351 non-hospitalized controls) corresponding to an overall success rate of 87% (95% CI: 84.8%–89.2%) that was similar in all the 4 groups of patients (*p* = 0.998) (S1 Table).

No significant differences were found regarding the age (*p* = 0.382) and sex (*p* = 0.468) distributions between cases and controls among ILI A(H1N1)pdm09 positive patients and ILI A(H1N1)pdm09 negative patients (Table 1). No significant differences were found between the analysed influenza positive hospitalized cases and influenza diagnosed hospitalized patients present in the NHD database, regarding age (*p* = 0.320) and sex (*p* = 0.133) (S2 Table).

**Table 1:** Patients characterization regarding age and gender variables.

	ILI A(H1N1)pdm09 positive patients			ILI A(H1N1)pdm09 negative patients		
	Hospitalized (cases)	Non-hospitalized (controls)	*p*	Hospitalized (cases)	Non-hospitalized (controls)	*p*
**Age (years)**						
Mean ± sd	16.6 ± 17.6	14.0 ± 12.4	0.815^1^	15.7 ± 20.2	13.5 ± 16.1	0.382^1^
Median (range)	10 (0–60)	9 (0–54)		4 (0–64)	6 (0–62)	
**Gender**						
% of women	40.5	46.2	0.382^2^	43.9	47.3	0.468^2^
(95% CI)	(30.0–51.0)	(39.0–53.4)		(36.5–51.3)	(42.1–52.5)	

^1^ *p-values* were obtained by the Wilcoxon test.

^2^ *p-values* were obtained by the Pearson's chi-squared test.

(CI, Confidence interval; *p*, p-value; sd, standard deviation).

Concerning *IFITM3* rs12252 variant characterization (Table 2), 3 patients carrying the CC genotype were detected (2 were ILI A(H1N1)pdm09 positive hospitalized patients and 1 was a ILI A(H1N1)pdm09 negative non-hospitalized patient).

**Table 2:** *IFITM3* rs12252 genotypic and allelic frequencies among the four groups of patients and its association with hospitalization, assuming a dominant model.

	ILI A(H1N1)pdm09 positive patients				ILI A(H1N1)pdm09 negative patients			
	Hospitalized (cases)	Non-hospitalized (controls)	OR Crude (95% CI)	OR Adjusted^2^ (95% CI)	Hospitalized (cases)	Non-hospitalized (controls)	OR Crude (95% CI)	OR Adjusted^2^ (95% CI)
**n**	84	184			173	351		
**Genotypes**								
**TT (%)**	73 (86.9)	152 (82.6)			134 (77.5)	312 (88.9)		
**CT (%)**	9 (10.7)	32 (17.4)			39 (22.5)	38 (10.8)		
**CC (%)**	2 (2.4)	0 (0)			0 (0)	1 (0.28)		
**Alleles**								
**C (%)**	13 (7.7)	32 (8.7)			39 (11.3)	40 (5.7)		
**T (%)**	155 (92.3)	336 (91.3)			207 (92.3)	662 (94.3)		
**Dominant model**^1^								
**CT/CC (%)**	11 (13.1)	32 (17.4)			39 (22.5)	39 (11.1)		
***vs***								
**TT (%)**	73 (86.9)	152 (82.6)			134 (77.5)	312 (88.9)		
			0.72	0.732			2.33	2.542
			(0.34–1.50)	(0.33–1.50)			(1.43–3.79)	(1.54–4.19)
***p value***			0.376	0.404			<0.001	<0.001

^1^ Other genetic models were not considered due the low frequency of CC genotype and the existence of zero patients with CC genotypes in 2 groups.

^2^Adjustment for age and gender by logistic regression.

Regarding A(H1N1)pdm09 infected patients, 10.7% (95% CI: 4.1–17.3) of the hospitalized cases and 17.4% (95% CI:11.9–22.9) of the non-hospitalized controls had the CT genotype. The hospitalized A(H1N1)pdm09 infected cases had a C allele frequency of 7.7% (95% CI: 3.6–11.8) while the non-hospitalized controls had a C allele frequency of 8.7% (95% CI: 5.8–11.6). In the A(H1N1)pdm09 negative patients, 22.5% (95% CI:16.3–28.8) of the hospitalized cases and 10.8% (95% CI: 7.6–14.1) of the non-hospitalized controls carried the CT genotype. The hospitalized cases have a C allele frequency of 11.3% (95% CI: 8.0–14.6) while the non-hospitalized controls presented a C allele frequency of 5.7% (95% CI: 2.2–9.2) (Table 2).

To assess if there is a higher risk of hospitalization for patients who carry the C allele both in the presence and absence of the A(H1N1)pdm09 virus infection, we performed a stratified analysis assuming a dominant model, as presented in Table 2. In the presence of A(H1N1)pdm09 virus infection, we observed that there is not a higher risk of being hospitalized for patients with the CT/CC genotype compared to the TT genotype (Adjusted Odds Ratio: 0.73 (95% CI: 0.33–1.50). However, when we use the same approach to assess if there is a higher risk of being hospitalized for the C allele carriers in patients negative for A(H1N1)pdm09 virus infection we found that the risk of being hospitalized among the CT/CC genotype carriers is significantly higher than the risk of being hospitalized in the TT genotype carriers (Adjusted Odds Ratio: 2.54 (95% CI: 1.54–4.19)).

## Discussion

In order to measure the association between the *IFITM3* rs12252 variant and influenza A(H1N1)pdm09 severity infection among ILI patients without reported comorbilities, we have designed a specific case-control study, in the H1N1 pandemic scenario. Our results show that the *IFITM3* rs12252 variant was associated with respiratory disease hospitalizations but not in the ILI patients with a laboratory confirmed Influenza A(H1N1)pdm09 infection. On the other hand, we observed that the risk of being hospitalized for the CT/CC genotype was 2.54 fold when compared to the TT genotype in ILI patients negative for influenza A(H1N1)pdm09 infection.

Given these results, we hypothesize that this variant is involved in the ILI symptoms severity associated with other respiratory infections. During the H1N1 pandemic influenza season, the Portuguese Laboratory Network just carried out the influenza virus detection (influenza A(H1N1)pdm09, influenza A(H1) seasonal, influenza A(H3) and influenza B virus), therefore we must consider the hypothesis of the presence of other infectious agents associated with respiratory disease (adenovirus, coronavirus, rhinovirus, respiratory syncytial virus, metapneumovirus, parainfluenza virus, *Haemophilus influenzae*, *Mycoplasma pneumoniae*, *Streptococcus pneumoniae* and other less frequent respiratory agents) in the Influenza negative samples that could explain our results. In addition, some studies show that *IFITM3* is also important in the mediation of other virus infections such as West Nile Virus and Dengue Virus [20] and this hypothesis could justify the higher percentage of C allele carriers in the ILI hospitalized Influenza negative cases. The future detection of other respiratory virus in the analyzed samples would be important to clarify our results.

When comparing our work with the study previously published by Everit *el al* (2012) [11] on this SNP regarding European patients, we found a similar percentage of C allele carriers among the influenza hospitalized positive cases (13.2% versus 13.1%). However, the comparison group were not ILI non hospitalized Influenza A(H1N1)pdm09 positive patients, meaning that they did not test the hypothesis that the *IFITM3* rs12252 variant was involved in the severity of the Influenza infection. More specifically, when comparing the general population without infection (European 1000 genomes) with the ILI hospitalized influenza positive patients they are evaluating both infection susceptibility and severity without the ability to distinguish each effect.

**Fig 2:**
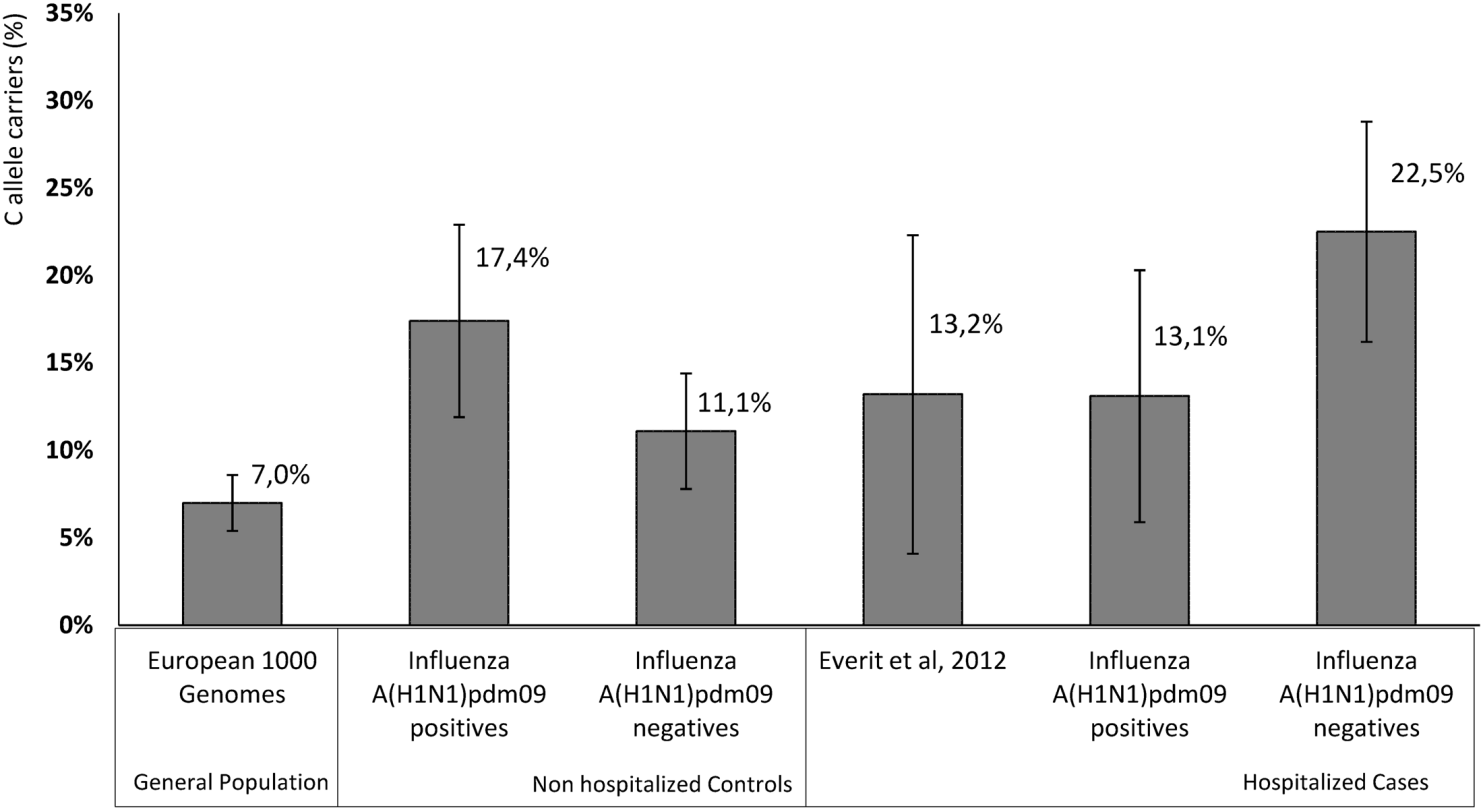
Percentage of C allele carriers (CC or CT genotypes) in the different samples. General population data are from the European 1000 genomes (Data from [11] were also included).

The percentage of the C allele carriers in the ILI non-hospitalized Influenza A(H1N1)pdm09 negative controls is the closest to the general population (European 1000 genomes), as expected (Fig 2).

Our results must be interpreted taking into account some limitations that are specific of the study design. The case definition (“hospitalization”) used to measure disease severity might not be considered as the best measure in a pandemic scenario given that less severe cases were likely to be hospitalized due to the initial pandemic alert, reducing the effect of *IFITM3* rs12252 on the risk of severity (hospitalization). However, we have reduced this bias by excluding patients with disease onset reported in the contention phase when health authorities had the indication to hospitalise all influenza A(H1N1)pdm09 cases. In addition, the hospitalization criteria that were used in the different health units might not be uniform and could be different from influenza A(H1N1)pdm09 positive and negative cases given the pandemic alert and the indications of the health authorities This differential criterion for hospitalization could explain the presence of the *IFITM3* rs12252 association in the negative influenza patients and the absence in the positive influenza patients. The extent of this bias could not be evaluated with the available data. On the other hand, we only performed the confounding adjustment for age and sex variables in the logistic regression analysis, but the reduction of possible confounding bias were also achieved by the exclusion criteria used in the patients selection, excluding patients with known risk factors for influenza infection complication. Regarding the possible selection bias, we were only able to evaluate the representativeness of the influenza hospitalized patients with less 65 years of age without known co morbidities, the patients recorded in the NHD during H1N1 pandemic were not statistical different from the ones included in the study regarding age and sex variables.

Due the low frequency of the *IFITM3* rs12252 observed in the Portuguese population and also in the European population (3%), a large number of cases would be needed to better define this association in our population. In the future, it will be important to perform an integrative approach to clarify why some healthy individuals resist infection or recover quickly, others experience severe disease associated with the influenza infection. It could be done by considering not only the virus-host genome interactions but also immunity, vaccination, weather conditions and other environmental factors that could help to clarify the present results. Moreover, early screening for the host genetic background could be a promising strategy to identify potential therapeutic targets and might help to evaluate and predict the severity of disease thereby providing critical information for decision making during treatment [21].

In conclusion, this study showed that the *IFITM3* rs12252 variant was associated with the hospitalization risk in the ILI hospitalized Influenza A(H1N1)pdm09 negative patients. However, the same association was not found in the ILI hospitalized Influenza A(H1N1)pdm09 positive cases and we cannot confirm that this variant is involved in the ILI symptoms severity associated with Influenza A(H1N1)pdm09 infection and the absence of the effect in this group could be due to bias associated with hospitalization criteria during the pandemic.

## Supporting Information

S1 Table Genotyping success rates in each ILI patients group. (DOCX)

S2 Table Age and sex comparison between the ILI hospitalized Influenza A(H1N1)pdm09 positive cases and the DRG patients. (DOCX)
