# Supplementary material for: Hospitalization Risk Due to Respiratory Illness Associated with Genetic Variation at IFITM3 in Patients with Influenza A(H1N1)pdm09 Infection: A Case-Control Study
Source: PLoS One. 2016 Jun 28;11(6):e0158181. doi: 10.1371/journal.pone.0158181 (PMC4924831; doi:10.1371/journal.pone.0158181)
Supplement: S1 Table — (DOCX) [file pone.0158181.s001.docx]

### Additional File 1

Table 1- Genotyping success rates in each ILI patients group.

|  | ILI A(H1N1)pdm09 positive patients | | ILI A(H1N1)pdm09 negative patients | | *p* |
| --- | --- | --- | --- | --- | --- |
|  | Hospitalized  (cases) | Non-hospitalized  (controls) | Hospitalized  (cases) | Non-hospitalized  (controls) |  |
| n | 96 | 212 | 198 | 403 | - |
| Genotyped samples  (%) | 84  (87.5%) | 184  (86.8%) | 173  (84.4%) | 351  (87.1%) | 0.998^1^ |

*^1^p-values* were obtained by the Pearson's chi-squared test.
