# Supplementary material for: Hospitalization Risk Due to Respiratory Illness Associated with Genetic Variation at IFITM3 in Patients with Influenza A(H1N1)pdm09 Infection: A Case-Control Study
Source: PLoS One. 2016 Jun 28;11(6):e0158181. doi: 10.1371/journal.pone.0158181 (PMC4924831; doi:10.1371/journal.pone.0158181)
Supplement: S2 Table — (DOCX) [file pone.0158181.s002.docx]

### Additional File 2

Table 1- Age and sex comparison between the ILI hospitalized Influenza A(H1N1)pdm09 positive cases and the DRG patients.

|  | ILI hospitalized Influenza A(H1N1)pdm09 positive cases | DRG patients | *p-value* |
| --- | --- | --- | --- |
| n | 84 | 587 |  |
| Age (years)  Mean ± sd  Median (range) | 16.6 ± 17.6  10 (0-60) | 15.3 ± 17.0  8 (0-64) | 0.320^1^ |
| Gender  % of women  (95% CI) | 40.5  (30.0-51.0) | 49.2  (45.1-53.4) | 0.133^2^ |

^1^ *p-values* were obtained by the Wilcoxon test. ^2^ *p-values* were obtained by the Pearson's chi-squared test.
